# Supplementary material for: CKII Control of Axonal Plasticity Is Mediated by Mitochondrial Ca2+ via Mitochondrial NCLX
Source: Cells. 2022 Dec 9;11(24):3990. doi: 10.3390/cells11243990 (PMC9777275; doi:10.3390/cells11243990)
Supplement: Supplementary file 1 [file cells-11-03990-s001.zip › cells-1980233-supplementary.pdf]

Supplemental Information

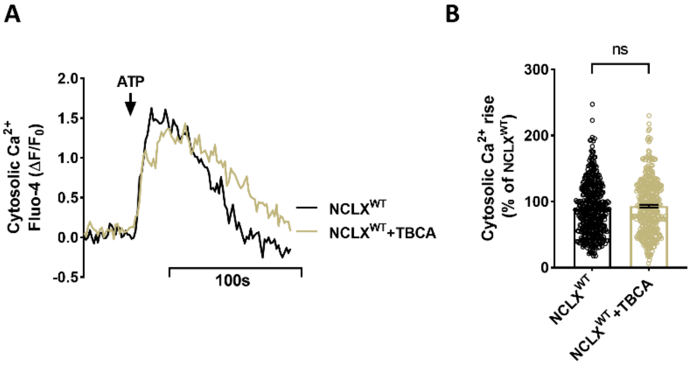

Figure S1. TBCA effect on Cytosolic Ca^{2+}

(A) Representative fluorescent traces of SH-SY5Y Cells co-expressing shNCLX and $NCLX^{WT}$ (black), treated with TBCA (10µM) for 2 hours (gold) and preloaded with Fluo-4 (2µM). Cytosolic Ca^{2+} transient was triggered by application of extracellular ATP (100µM) via Ca^{2+}-free Ringer's solution.

(D) Quantification of cytosolic Ca^{2+} influx amplitude in (A) (unpaired two-tailed t-test, t=1.358, df=864: ns; n= $NCLX^{WT}$ (452), $NCLX^{WT}$+TBCA (414)).

ns- Not significant. Error bars denote SEM.

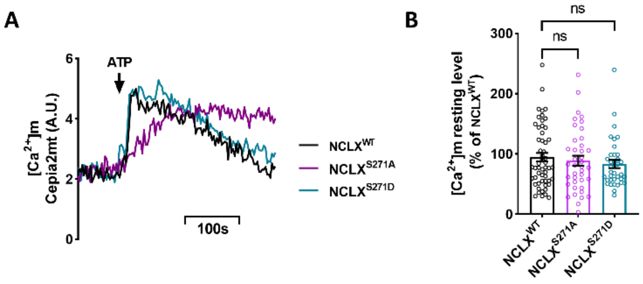

Figure S2

(A) Representative fluorescent (non-normalized) traces of ATP (100µM) induced $[Ca^{2+}]m$ transients in Cepia2mt expressing SH-SY5Y cells co-expressing shNCLX and $NCLX^{WT}$ (black) or mutant $NCLX^{S271A}$ (purple)\ $NCLX^{S271D}$ (turquoise). A.U.- arbitrary units

(B) Quantification of $[Ca^{2+}]m$ basal intensity in (A) (Welch and Brown-Forsythe ANOVA, F(2,101.4)= 0.8545; Dunnett's T3 multiple comparisons test; $NCLX^{WT}$ vs. $NCLX^{S271A}$: ns; $NCLX^{WT}$ vs. $NCLX^{S271D}$: ns; n= $NCLX^{WT}$ (54), $NCLX^{S271A}$ (40), $NCLX^{S271D}$ (37)).

ns- Not significant. Error bars denote SEM.
